# Propranolol 0.2% Eye Micro-Drops for Retinopathy of Prematurity: A Prospective Phase IIB Study

**DOI:** 10.3389/fped.2019.00180

**Published:** 2019-05-07

**Authors:** Luca Filippi, Giacomo Cavallaro, Elettra Berti, Letizia Padrini, Gabriella Araimo, Giulia Regiroli, Genny Raffaeli, Valentina Bozzetti, Paolo Tagliabue, Barbara Tomasini, Annalisa Mori, Giuseppe Buonocore, Massimo Agosti, Angela Bossi, Gaetano Chirico, Salvatore Aversa, Pina Fortunato, Silvia Osnaghi, Barbara Cavallotti, Martina Suzani, Maurizio Vanni, Giulia Borsari, Simone Donati, Giuseppe Nascimbeni, Daniel Nardo, Stefano Piermarocchi, Giancarlo la Marca, Giulia Forni, Silvano Milani, Ivan Cortinovis, Maura Calvani, Paola Bagnoli, Massimo Dal Monte, Anna Maria Calvani, Alessandra Pugi, Eduardo Villamor, Gianpaolo Donzelli, Fabio Mosca

**Affiliations:** ^1^Neonatal Intensive Care Unit, Medical Surgical Fetal-Neonatal Department, “A. Meyer” University Children's Hospital, Florence, Italy; ^2^Neonatal Intensive Care Unit, Fondazione IRCCS Ca' Granda Ospedale Maggiore Policlinico, Milan, Italy; ^3^Department of Clinical Sciences and Community Health, University of Milan, Milan, Italy; ^4^Neonatal Intensive Care Unit, MBBM Foundation, San Gerardo Hospital, Monza, Italy; ^5^Neonatal Intensive Care Unit, University Hospital of Siena, Policlinico Santa Maria alle Scotte, Siena, Italy; ^6^Department of Molecular and Developmental Medicine, University of Siena, Siena, Italy; ^7^Neonatal Intensive Care Unit, Del Ponte Hospital, Varese, Italy; ^8^Neonatal Intensive Care Unit, Children's Hospital, University Hospital “Spedali Civili” of Brescia, Brescia, Italy; ^9^Pediatric Ophthalmology, “A. Meyer” University Children's Hospital, Florence, Italy; ^10^Department of Ophthalmology, Fondazione IRCCS Ca' Granda, Ospedale Maggiore Policlinico, Università degli Studi di Milan, Milan, Italy; ^11^Department of Ophthalmology, ASST Monza, San Gerardo Hospital, Monza, Italy; ^12^Pediatric Ophthalmology, University Hospital of Siena, Policlinico Santa Maria alle Scotte, Siena, Italy; ^13^Department of Surgical and Morphological Sciences, Section of Ophthalmology, University of Insubria, Varese, Italy; ^14^Department of Ophthalmology, University Hospital “Spedali Civili” of Brescia, Brescia, Italy; ^15^Department of Women's and Children's Health, Azienda Ospedaliera di Padova, University of Padua, Padua, Italy; ^16^Department of Ophthalmology, University of Padua, Padua, Italy; ^17^Newborn Screening, Biochemistry and Pharmacology Laboratory, Meyer Children's University Hospital, Florence, Italy; ^18^Laboratory “G.A. Maccacro,” Department of Clinical Sciences and Community Health, University of Milan, Milan, Italy; ^19^Oncohematology Unit, Department of Pediatric Oncology, “A. Meyer” University Children's Hospital, Florence, Italy; ^20^Department of Biology, Unit of General Physiology, University of Pisa, Pisa, Italy; ^21^Department of Pharmacy, “A. Meyer” University Children's Hospital, Florence, Italy; ^22^Clinical Trial Office, “A. Meyer” University Children's Hospital, Florence, Italy; ^23^Department of Pediatrics, Maastricht University Medical Center, School for Oncology and Developmental Biology, Maastricht, Netherlands

**Keywords:** propranolol, beta blocker, proliferative retinopathy, angiogenesis, preterm newborn

## Abstract

**Background:** Oral propranolol reduces retinopathy of prematurity (ROP) progression, although not safely. Propranolol 0.1% eye micro-drops administered to newborns with stage 2 ROP are well-tolerated, but not sufficiently effective.

**Methods:** A multi-center open-label trial was conducted to assess the safety and efficacy of propranolol 0.2% eye micro-drops in newborns with stage 1 ROP. The progression of the disease was evaluated with serial ophthalmologic examinations. Hemodynamic, respiratory, biochemical parameters, and propranolol plasma levels were monitored. Demographic and perinatal characteristics, co-morbidities and co-intervention incidences, together with ROP progression, were compared with a historical control group in the same centers participating in the trial.

**Results:** Ninety-eight newborns were enrolled and compared with the historical control group. Populations were not perfectly homogeneous (as demonstrated by the differences in the Apgar score and the different incidence rate in surfactant administration and oxygen exposure). The progression to ROP stage 2 or 3 plus was significantly lower than the incidence expected on the basis of historical data (Risk Ratio 0.521, 95% CI 0.297– 0.916). No adverse effects related to propranolol were observed and the mean propranolol plasma level was significantly lower than the safety cut-off of 20 ng/mL. Unexpectedly, three newborns treated with oral propranolol before the appearance of ROP, showed a ROP that was unresponsive to propranolol eye micro-drops and required laser photocoagulation treatment.

**Conclusion:** Propranolol 0.2% eye micro-drops were well-tolerated and appeared to reduce the ROP progression expected on the basis of a comparison with a historical control group. Propranolol administered too early appears to favor a more aggressive ROP, suggesting that a β-adrenoreceptor blockade is only useful during the proliferative phase. Further randomized placebo-controlled trials are required to confirm the current results.

**Clinical Trial Registration**

The trial was registered at ClinicalTrials.gov with Identifier NCT02504944 and with EudraCT Number 2014-005472-29.

## Introduction

Despite remarkable progress in many areas of neonatal medicine, retinopathy of prematurity (ROP) remains a very common and disabling complication of premature birth (1). Even though ROP is currently considered a multi-factorial disease that involves both oxygen-dependent and oxygen-independent factors such as nutritional intakes (2), phenomena such as low gestational age (GA), low birthweight, and supplemental oxygen therapy are the main factors associated with it (3). Currently, the possibility to prevent or slow down the progression of ROP is predominantly dependent on a controlled and careful use of oxygen, but the possibility of pharmacological interventions arouses great interest (4).

ROP is a typical oxygen-dependent biphasic disease, where what happens in the first phase, following oxygen exposure, apparently mirrors what happens during the second phase, when the retina becomes hypoxic (3). In the hypoxic uterine environment, retinal vascularization begins from the 16th week of gestation, and is completed at ~40 weeks of GA. Very preterm newborns are born with peripheral retinae that are still avascular. The exposure to a hyperoxic environment induces the down-regulation of retinal pro-angiogenic factors, such as VEGF, which contributes to growth arrest, or even regression, of the normal retinal vasculature. This is the first phase of ROP, the ischemic phase, where vascular obliteration favors the transition to the hypoxic phase. In fact, as the infant matures, the avascular retina becomes increasingly hypoxic due to the discrepancy between the increased metabolic demands of the maturing retina and the rudimentary vascularization. This hypoxia induces a localized up-regulation of proangiogenic factors, leading to the second phase of ROP, the proliferative phase, characterized by vaso-proliferation and retinal neovascularization. The degree of vascular obliteration during the ischemic phase affects the severity of retinal hypoxia, which subsequently regulates the production of retinal VEGF in the proliferative phase (3, 5).

Recently, we have demonstrated in the murine model of oxygen-induced retinopathy (OIR), the model usually adopted to experimentally reproduce ROP (6), that the β-adrenergic system plays a central role in the promotion of retinal neovascularization. In fact, the retinal ischemia induced by oxygen exposure promotes the release of norepinephrine, which reacts with β2 and β3- adrenoreceptors (β-AR) inducing the up-regulation of hypoxia-induced factor (HIF), vascular endothelial growth factor (VEGF) and insulin growth factor (IGF-1). Systemic propranolol, administered in mice during the proliferative phase of OIR, reduces its progression thanks to the down-regulation of such proangiogenic factors (7–9). Pilot clinical trials have verified that oral propranolol administered to newborns with ROP slows down its progression, reducing the need for laser photocoagulation or anti-VEGF treatment (10–13). However, this treatment aroused serious safety concerns because unstable preterm newborns receiving 1–2 mg/kg/day, whose mean propranolol plasma concentrations were between 20 and 60 ng/mL, showed life-threatening adverse events. A series of apneas, bradycardias, and hypotensions were observed in newborns simultaneously with the development of an infection, or during the induction of general anesthesia, or after respiratory failure due to massive atelectasia or bronchospasm (10). Some of these infants were refractory to adrenaline (probably due to the β-ARs blockade), and recovered after receiving terlipressin (10). This experience led to the exploration of topical administration of propranolol as a possible safer approach: the objective was to obtain an efficacy similar to what we had observed with oral propranolol, but with plasma concentrations lower than 20 ng/mL, which is considered a sort of safe cut-off value. This strategy was evaluated initially in animal models, and then in newborns. In mice with OIR, propranolol eye-drops confirmed its efficacy in counteracting retinal neovascularization (14). In rabbits, the administration of propranolol 0.1% eye-drops dramatically increased the retina/plasma ratio compared with oral administration, suggesting that it was possible to conciliate ocular efficacy with low plasma levels (15). The first study, performed in newborns with stage 2 ROP, showed that propranolol 0.1% eye micro-drops were well-tolerated, but not sufficiently effective in reducing ROP progression (16). However, the low plasma propranolol levels and the lack of adverse effects encouraged the administration of a higher concentration (0.2%) to newborns at an earlier stage of the disease (ROP stage 1) (17).

## Methods

### Preliminary Analysis

To plan the present multi-center, open-label, single arm, phase IIB trial, a preliminary analysis was performed to evaluate the historical natural evolution of ROP in the neonatal intensive care units (NICUs) involved in the trial from 2011 to 2015. The study protocol has previously been published (17). Demographic and perinatal characteristics of the historical cohort belonging to the six NICUs originally participating in the trial and the percentage of progression from stage 1 ROP at first diagnosis have also been published (17). However, a seventh Center was added to the study and the retrospective analysis was updated. Results of this analysis are reported in Results section.

### Definitions

In order to establish the location and severity of ROP, standard international ROP classification was adopted (18).

### Patients

From August 2015 to April 2018, preterm newborns with GA ≤ 32 weeks and birthweight ≤ 1500 g, diagnosed with stage 1 ROP in zone II or III, who were admitted to the NICUs taking part in the study (University Hospital of Florence, Milan, Monza, Siena, Varese, Brescia, and Padua) were enrolled in this trial. Newborns with heart failure, severe and persistent hypotension, persistent bradycardia (heart rate <90 beats per minute), second or third-degree atrioventricular blocks, congenital cardiovascular anomalies (except for persistent ductus arteriosus, patent foramen ovale, and small ventricular septal defects), renal failure, current cerebral hemorrhage, and other diseases that contraindicate the use of β-AR blockers on enrolment were excluded from the study. Moreover, newborns with ROP at a more advanced stage than stage 1 at first diagnosis and with aggressive posterior ROP (AP-ROP) were also excluded.

### Intervention

Treatment with ocular propranolol 0.2 % solution was started immediately after stage 1 ROP was diagnosed. The eye solution was prepared sterilely by diluting propranolol hydrochloride powder (ACEF, Fiorenzuola d'Arda, Piacenza, Italy), in sterile water for injection at a concentration of 2%. Then, the propranolol 0.2% solution was obtained in a horizontal laminar flow hood, adding 9 ml of saline solution to 1 ml of propranolol 2% preparation (15, 17). Newborns received three micro-drops of 6 μL (12 μg propranolol per micro-drop) in each eye, applied with a micropipette, every 6 h. The nurses and the parents of infants nearing discharge were instructed on how to use the micropipette to also administer the micro-drops at home. After each administration, the nasolacrimal duct was carefully compressed for 30 s to decrease the drug absorption through the conjunctival and nasal vessels. Treatment was continued until retinal vascularization was complete, but for no longer than 90 days.

While infants were receiving supplemental oxygen, the target range of oxygen saturation was maintained between 88 and 93%. The ophthalmologic approach was in accordance with the guidelines adopted by the Early Treatment for Retinopathy of Prematurity (ETROP) Cooperative Group and international guidelines (19–21). Therefore, newborns with ROP who progressed to stage 2 plus or 3 plus were treated with laser photocoagulation or intravitreal anti-VEGF administration (19). The ophthalmologists chose the treatment they considered most appropriate.

### Safety

Hemodynamic parameters, diuresis and respiratory parameters were continuously monitored during the first 3 weeks of treatment. Bradycardia was defined as a single heart-rate drop below 100 bpm; apnea was defined as a pause in breathing for more than 20s or less when associated with desaturation, bradycardia, pallor, or reduced tone. Hypotension was defined as mean arterial blood pressure less than the 10th centile for gestation/birth weight and postnatal age (16). A complete blood count, serum electrolytes levels, and renal and liver function tests were performed before starting treatment (T0) and once a week for the first 3 weeks of treatment (T7, T14, and T21). An electrocardiogram and echocardiogram were performed before starting treatment and once a week for the next 3 weeks. Any drugs that were concomitantly administered were recorded.

### Plasma Propranolol Evaluation

The plasma propranolol level was measured in dried blood spots with the liquid-chromatography tandem-mass spectrometry method (22) at the steady state (10th day of treatment), before administering therapy (T0), after 2 (T2), 4 (T4), and 6 h (T6) (23). The coefficient of variation (CV), a measure of relative variability, was calculated as the ratio of the standard deviation to the mean propranolol values, for each point (from T0 to T6).

### Efficacy

The primary outcome was the rate of progression of the disease to ROP stage 2 or 3 plus. Complete ophthalmologic evaluations were performed in accordance with international guidelines (21). The ophthalmologic exam verified the absence of local adverse events due to treatment with propranolol eye micro-drops, and also monitored the ROP progression using indirect ophthalmoscopy. The RetCam Imaging System was systematically used by ophthalmologists to evaluate ROP evolution.

### Stop Criteria and Dose Changes

A strategy to follow in the event of any severe adverse events attributable to topical propranolol, or in the case of mean propranolol plasma concentrations being higher than 20 ng/mL, was planned (17). Considering the high risk of adverse events after propranolol administration in unstable newborns, in the case of severe adverse events, ascribable to propranolol, the treatment was to be promptly interrupted and the newborn excluded from the study. In the case of surgery and/or anesthesia, the propranolol eye micro-drops treatment was to be discontinued for at least 24 h. Then, the study was to recommence by reducing the dosage to two micro-drops four times daily in each eye. Similarly, the study was to recommence with an increase in the concentration of propranolol up to 0.3% in the case of treatment failure in terms of efficacy during the first stage of the study, but only if plasma propranolol concentrations were below the cut off of 20 ng/ml (17).

### Ethical Approval and Informed Consent

The study was conducted in accordance with the recommendations of Good Clinical Practice. The study was approved by the Italian Medicines Agency Institutional Review Board and the Ethics Committees of all the participating centers. Written informed consent was obtained from the parents of the newborns in accordance with the Declaration of Helsinki.

### Statistical Analysis

**Figure 1:**
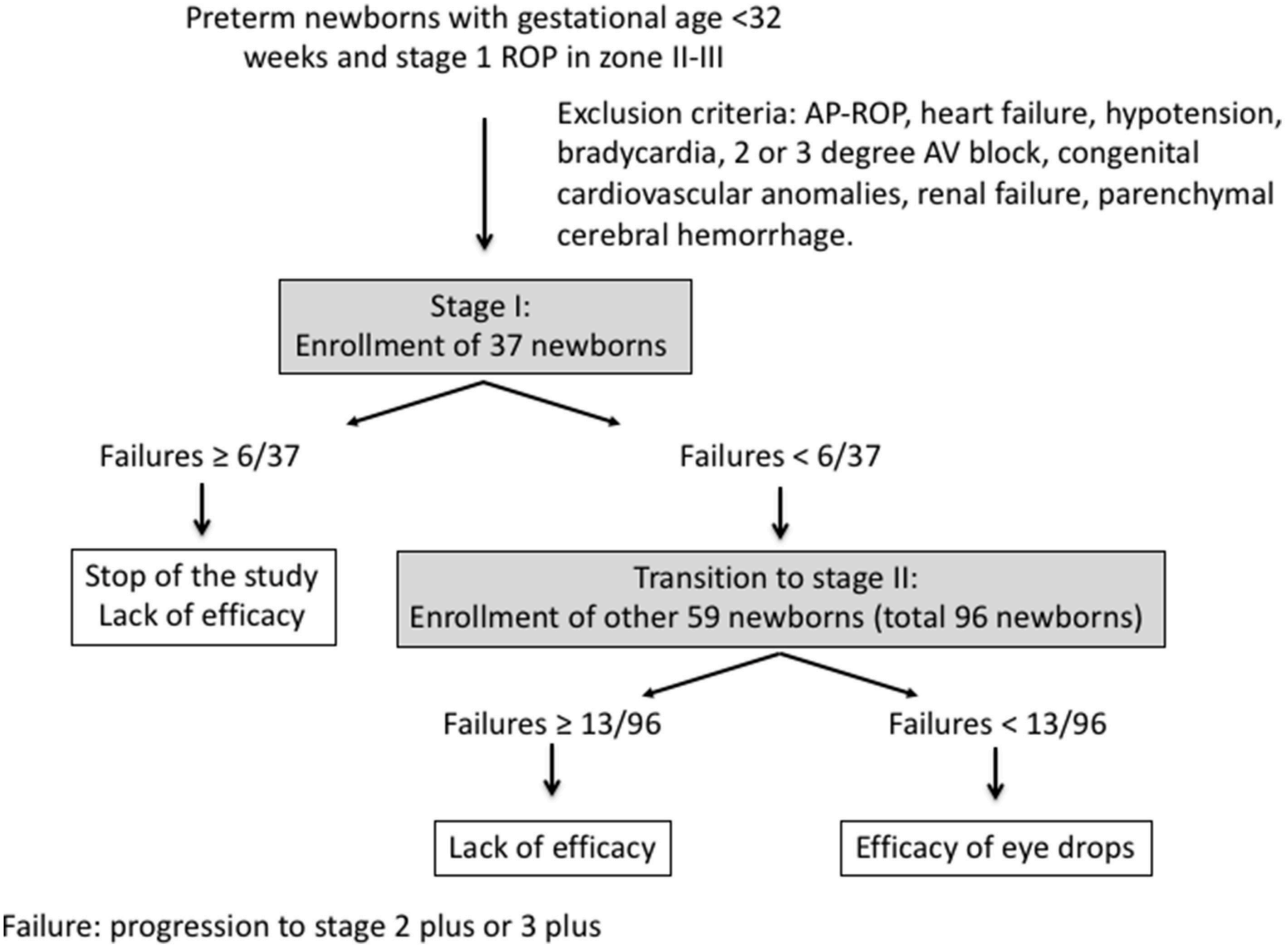
The Simon two-stage optimal design algorithm of the study.

This study was planned according to the Simon optimal two-stage design for phase II clinical trials (24). The historical progression rate from stage 1 ROP to stage 2 plus or 3 plus in the NICUs involved in the trial was 23.7%, but we maintained the estimate of 19% that was obtained in a first analysis and had been used to get authorization from the Italian Medicines Agency (17). The treatment was considered effective if it halved this progression ratio. Hence, considering an alpha error of 0.05 and a power of 80%, the study analysis plan required 37 participants to be enrolled in Stage 1, with <6 participants meeting failure criteria in Stage 1 before Stage 2 was conducted. If Stage 1 criteria was not met, the study would be terminated for lack of efficacy. If <6 participants met Stage 1 failure criteria, 59 additional participants would be enrolled in Stage 2. A total of <13 failures of the 96 total participants (in both Stages) would indicate sufficient promise to warrant further investigation (Figure 1). Moreover, the study was to be considered a failure if the mean propranolol plasma concentration at the steady-state was >20 ng/mL.

Categorical variables were expressed as counts or percentages and compared using the chi-square test. Continuous variables were expressed as mean (SD) if they followed a normal distribution and if they were compared using an unpaired, two-sided *t*-test. If not normally distributed, continuous variables were expressed as median values (range) and compared using the Mann-Whitney *U*-test. The Kolmogorov-Smirnov test was used to test for normal distribution of continuous data. The null hypothesis was accepted with a *P* > 0.05. The efficacy of the treatment was evaluated by means of the risk ratio (RR), the ratio between the proportion of subjects progressing to more advanced-stage ROP in the propranolol group vs. the historical control group. In the case of a statistically significant reduction in the RR, the number of needed to treat (NNT) was calculated.

## Results

### Historical Control Group

In the period 2011–2015, 2,746 very low birth weight (VLBW) newborns were admitted to the seven NICUs participating in the trial. The number of newborns with stage 1 ROP at first diagnosis was 333 (12.1%), and the number of infants with stage 2 plus or 3 plus was 79 (23.7% of newborns with stage 1 ROP at first diagnosis and 2.9% of all VLBW newborns). These newborns represent the historical control group.

### Treated Group

Overall, among the 1,131 VLBW newborns admitted, 105 were eligible for the study. However, 7 newborns were excluded: 5 newborns because they had developed AP-ROP, 1 newborn for protocol deviation, and 1 newborn because their parents withdrew their consent to proceed with the treatment after 4 days after enrolment. No newborn was either excluded from the study nor removed during the study for cardiac reasons. Electrocardiographic evaluation showed a right bundle branch block in two infants at enrolment. Cardiac ultrasounds revealed a ventricular septal defect that was hemodynamically not relevant in two patients, and a moderate left ventricular hypertrophy in one infant at the enrolment stage.

A total of ninety-eight newborns were enrolled and received propranolol 0.2% eye micro-drops (treated group). Treatment was started at a median of 54.5 (range 20–94) days of life (at a mean post-menstrual age of 34.2 ± 2.3 weeks), as soon as stage 1 ROP was diagnosed. Enrolment was performed ~2 weeks after the first eye examination, which occurred at a mean post-menstrual age of 31.6 ± 1.1 weeks. The demographic and perinatal characteristics of the study population are summarized in Table 1 and compared with the historical control group. Apgar scores were slightly but significantly higher in the treated group. A significantly reduced administration of surfactant, but a more prolonged oxygen supply (mean 67 ± 60 days vs. 53 ± 48 days, *p* = 0.037) were both observed in the treated group (Table 1).

**Table 1:** Demographic, perinatal characteristics, co-morbidities, and co-interventions of treated and historical cohorts.

**Demographic and perinatal characteristics**	**Treated group**	**Historical control group**	***p***
Newborns*, n*	98	333	
Gestational age, weeks, *mean ± SD*	26.7 ± 2.0	26.4 ± 2.1	0.198
Birth weight, g, *mean ± SD*	877 ± 250	843 ± 234	0.240
Male, *n (%)*	48 (49)	180 (52.9)	0.501
Cesarean delivery, *n (%)*	72 (73.5)	254 (76.3)	0.570
Stained amniotic fluid, *n (%)*	7 (7.14)	16 (4.8)	0.366
Apgar Score, 1 min, *median (range)*	5 (1–9)	5 (0–9)	0.014
Apgar Score, 5 min, *median (range)*	8 (4–10)	8 (0–10)	0.013
Post menstrual age at enrolment, weeks, *mean ± SD*	34.2 ± 2.3	34.1 ± 2.8	0.467
**CO-MORBIDITIES AND CO-INTERVENTION**			
Respiratory distress syndrome, *n (%)*	93 (94.9)	324 (97.3)	0.240
Surfactant treatment, *n (%)*	78 (79.6)	292 (87.7)	0.043
Duration of oxygen exposure (days), *median (range)*	58 (0–458)	44.0 (0–291)	0.047
Bronchopulmonary dysplasia^a^, *n (%)*	64 (65.3)	202 (60.7)	0.407
Candida sepsis, *n (%)*	5 (5.1)	12 (3.6)	0.504
Other sepsis, *n (%)*	50 (51)	174 (52.2)	0.831
Number of red blood cell transfusions, *median (range)*	5 (0–21)	5 (0–21)	0.333
Intraventricular hemorrhage, grade 3–4, *n (%)*	18 (18.4)	48 (14.4)	0.341
Post-hemorrhagic hydrocephalus, *n (%)*	7 (7.1)	17 (5.1)	0.441
Cholestasis, *n (%)*	26 (26.5)	61 (18.3)	0.075
Necrotizing enterocolitis, *n (%)*	9 (9.2)	34 (10.2)	0.766
Gastrointestinal perforation, *n (%)*	8 (8.2)	31 (9.3)	0.729
Surgical closure of patent ductus arteriosus, *n (%)*	18 (18.4)	55 (16.5)	0.668
Survival, *n (%)*	95 (96.9)	329 (98.8)	0.201

a * Oxygen requirement at 36 weeks' postmenstrual age*.

The median duration of treatment was 70 (range 12–90) days. No protracted interruption of treatment was reported, except for brief suspensions for surgical procedures. Three of the ninety-eight newborns had been treated with 2 mg/kg/day of oral propranolol before ROP development (two for cardiac hypertrophy and one for supra-ventricular tachycardia) and were then enrolled at the development of ROP stage 1.

### Safety

During the study, none of the severe adverse events that are usually related to propranolol (i.e., bradycardia, bronchospasm, severe hypotension) or severe local signs were observed. One newborn showed retinal hemorrhage in the right eye after 40 days of treatment, which appeared the day after mask ventilation for apnea. Treatment was not discontinued because a correlation with propranolol was considered unlikely. The following month, a bilateral retinal pallor was noticed. Treatment was discontinued after 90 days, and ROP regressed. Forty days after propranolol eye drops discontinuation, a bilateral cataract was observed.

Three infants died from causes unrelated to propranolol eye drops administration. One infant, suffering from chronic myelomonocytic leukemia, died from multi-organ failure concomitant with sepsis by Klebsiella Pneumoniae 52 days after the discontinuation of propranolol treatment. A second infant died from suspected viral sepsis 69 days after the discontinuation of propranolol treatment. A third infant, suffering from multiple thromboembolism, died because of renal failure after 24 days of treatment, with ROP stable at stage 2, but before ROP had completed its evolution, and therefore he was excluded from the outcome analysis.

**Figure 2:**
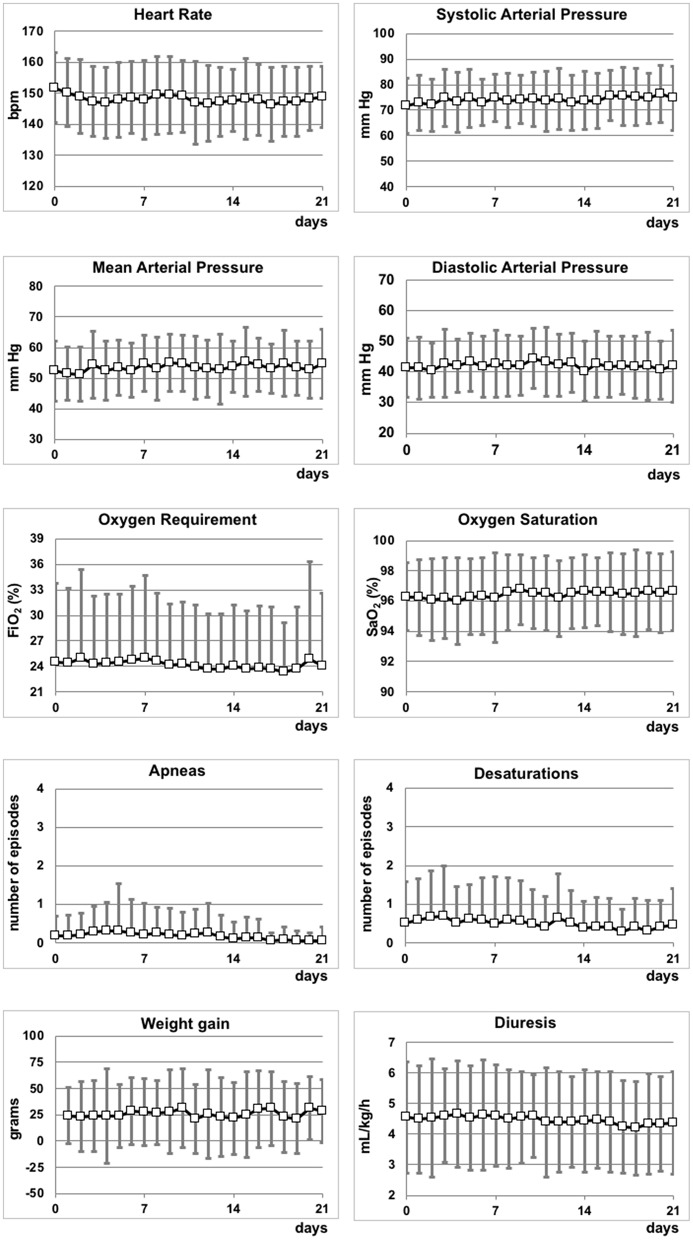
Hemodynamic and respiratory parameters, weight gain and diuresis during the first 21 days of the study.

Hemodynamic and respiratory parameters, weight gain and diuresis, continuously monitored during the first 21 days of treatment, did not present relevant abnormalities (Figure 2), and were in line with values observed after the administration of 0.1% propranolol eye micro-drops (16). Regarding the biochemical parameters that were monitored during the study, no substantial differences were observed between the values at the enrolment stage and the values after 7, 14, or 21 days. The decrease of total and conjugate bilirubin was compatible with a progressive improvement of the liver function. The only effect probably related to propranolol was the moderate increase of potassium values (also observed in a previous study) (10), although all values were within the normal range (Table 2).

**Table 2:** Biochemical parameters during the study of treated group.

	**Enrolment**	**7 days**	***P***	**14 days**	***P***	**21 days**	***P***
pH	7.36 ± 0.05	7.36 ± 0.06	0.746	7.36 ± 0.06	0.854	7.37 ± 0.05	0.099
pCO_2_, mmHg, *mean ± SD*	45.5 ± 8.2	48.2 ± 10.2	0.053	46.8 ± 9.0	0.341	46.9 ± 9.4	0.321
pO_2_, mmHg, *mean ± SD*	48.5 ± 18.5	49.1 ± 18.7	0.840	48.7 ± 20.1	0.937	47.8 ± 19.5	0.812
Base Excess, mmol/L, *mean ± SD*	0.3 ± 4.1	0.6 ± 4.2	0.585	0.6 ± 3.7	0.650	1.2 ± 4.1	0.153
${\text{HCO}}_{3}^{-}$, mmol/L, *mean ± SD*	25.3 ± 3.9	26.2 ± 4.3	0.107	26.0 ± 3.8	0.211	26.5 ± 4.3	0.040
White blood cells, × 10^9^/L, *mean ± SD*	11081 ± 5651	11170 ± 5480	0.963	10614 ± 6367	0.518	10386 ± 5107	0.339
Hemoglobin, g/dL, *mean ± SD*	10.3 ± 1.9	10.5 ± 1.8	0.619	10.4 ± 1.6	0.762	9.9 ± 1.7	0.088
Hematocrit, %, *mean ± SD*	30.1 ± 5.4	31.1 ± 5.4	0.270	30.8 ± 4.7	0.368	29.4 ± 4.0	0.306
Platelets, × 10^9^/L, *mean ± SD*	329 ± 139	340 ± 138	0.603	343 ± 129	0.506	347 ± 143	0.425
Glucose, mg/dL, *mean ± SD*	84 ± 22	81 ± 18	0.372	84 ± 19	0.968	83 ± 18	0.792
Sodium, mEq/L, *mean ± SD*	138 ± 3.2	138 ± 3.3	0.928	139 ± 3.7	0.124	139 ± 3.1	0.219
Potassium, mEq/L, *mean ± SD*	4.4 ± 0.7	4.5 ± 0.8	0.296	4.6 ± 0.8	0.069	4.8 ± 0.9	0.002
Chloride, mEq/L, *mean ± SD*	103 ± 5.4	102 ± 5.5	0.439	103 ± 5.5	0.648	103 ± 5.0	0.502
Total protein, g/L, *mean ± SD*	4.6 ± 0.6	4.7 ± 0.5	0.946	4.6 ± 0.5	0.701	4.7 ± 0.4	0.878
Urea nitrogen, mg/dL, *mean ± SD*	20 ± 15	21 ± 19	0.635	21 ± 26	0.690	22 ± 19	0.529
Creatinine, mg/dL, *mean ± SD*	0.39 ± 0.3	0.39 ± 0.4	0.848	0.36 ± 0.3	0.464	0.30 ± 0.1	0.008
Aspartate transaminase, U/L, *mean ± SD*	46 ± 42	48 ± 39	0.770	50 ± 56	0.848	38 ± 22	0.121
Alanine transaminase, U/L, *mean ± SD*	31 ± 33	30 ± 29	0.906	31 ± 36	0.935	26 ± 23	0.326
Total bilirubin, mg/dL, *mean ± SD*	2.2 ± 3.0	1.8 ± 2.3	0.311	1.4 ± 2.1	0.057	1.1 ± 1.2	0.004
Conjugate bilirubin, mg/dL, *mean ± SD*	1.6 ± 2.8	1.3 ± 2.1	0.555	1.2 ± 2.1	0.458	0.6 ± 0.8	0.018
C-reactive protein, mg/dL, *mean ± SD*	0.69 ± 1.6	0.60 ± 1.2	0.694	0.87 ± 2.6	0.605	0.61 ± 1.7	0.784

*T-test was evaluated between values at enrolment and values after 7, 14, or 21 days of treatment*.

Newborns who were clinically stable after the first 3 weeks of treatment, were discharged and continued treatment at home. No extended hospital stays were observed for newborns involved in the study.

### Plasma Propranolol Level

**Figure 3:**
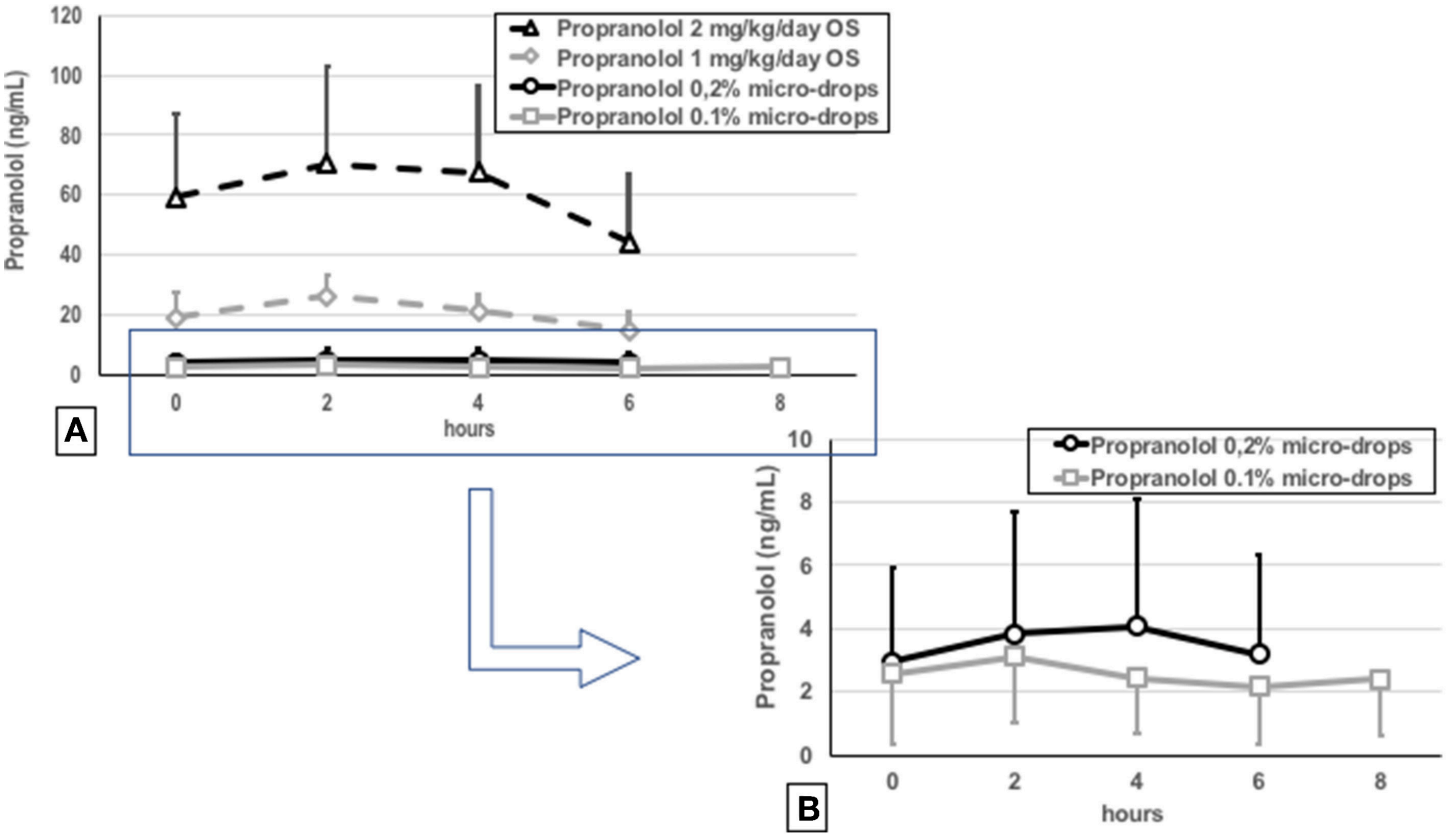
Plasma propranolol concentrations measured by dried blood spots in the newborns treated with 0.2% eye micro-drops (*continuous black line*), in the tenth day of treatment, compared with plasma concentrations obtained in the previous studies after 0.1% eye micro-drops (*continuous gray line)* (15), oral 1 mg/kg/day (*dotted gray line*) or oral 2 mg/kg/day (*dotted gray line*) propranolol administration **(A)** (9). Magnification of results observed with 0.2 and 0.1% eye micro-drops **(B)**.

Propranolol dried blood spots were obtained from 91 of the 98 patients. Large inter-individual differences in plasma concentrations were observed between the patients, and 20 infants showed plasma propranolol values steadily <2.5 ng/mL. The CV of propranolol values was very high (0.77 at T_0_, 0.82 at T_2_, 0.88 at T_4_, and 0.77 at T_6_). The mean plasma propranolol level on the 10th day (steady-state) was consistently below the cut-off value of 20 ng/mL (Figure 3). These values were ~50–80% higher than the values obtained with eye drops at 0.1% (16), and approximately 5 times lower than that reported after oral administration of 1 mg/kg/day of propranolol (10). Only one infant showed propranolol values higher than 20 ng/mL (27 ng/mL) after 2 h of eye micro-drops administration.

### Efficacy

After the enrolment of the first 37 infants, only 2 patients progressed to ROP stage 2 or 3 plus, and the mean plasma propranolol level was around 3–4.2 ng/mL. Therefore, the study progressed to the second stage, and all the planned newborns were enrolled. The analysis of the ophthalmologic outcome was performed for the 97/98 newborns that completed treatment.

**Figure 4:**
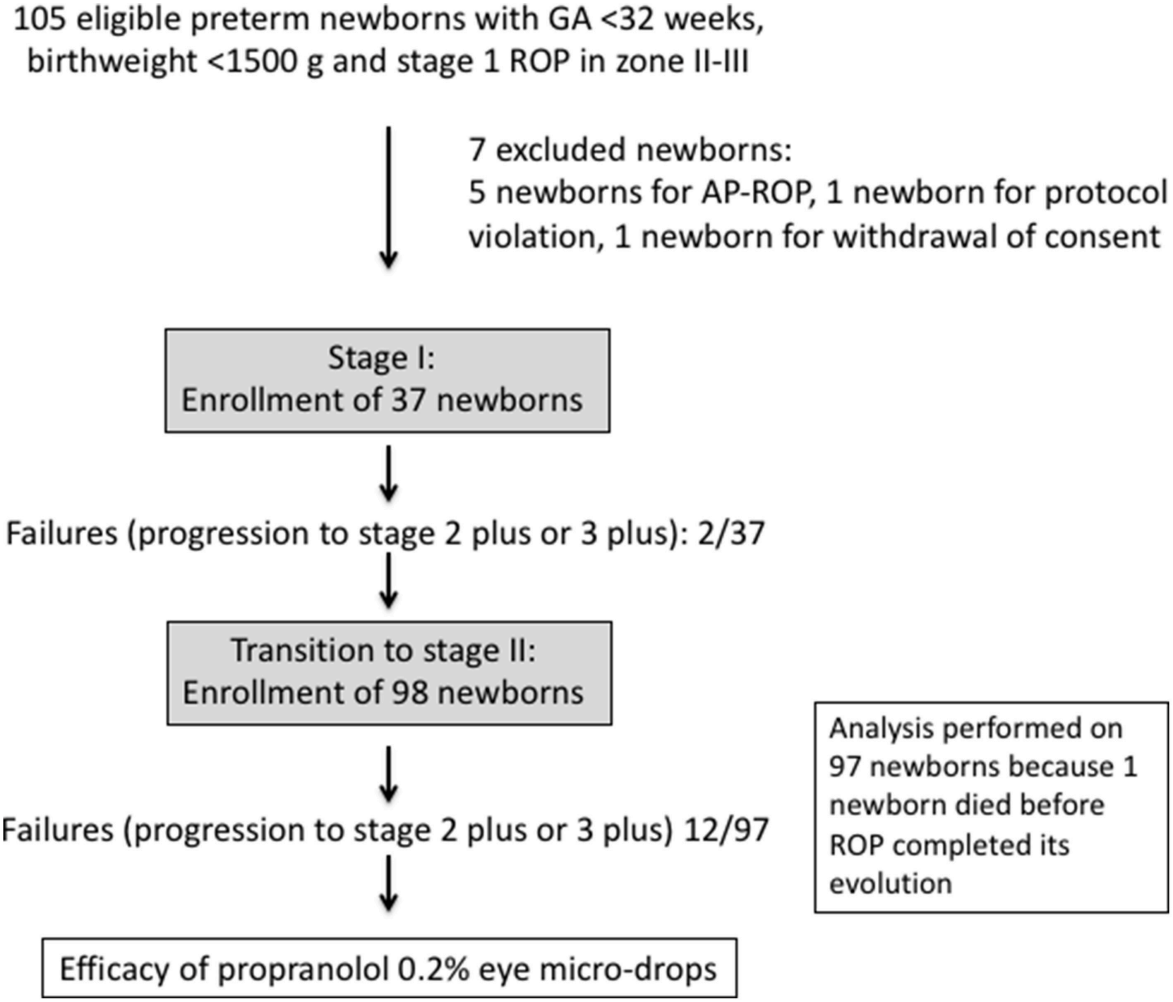
Schematic representation of the results of the trial.

Overall, 12/97 newborns (12.4%) progressed to stage 2 plus or 3 plus (six to stage 2 plus and six to stage 3 plus), approximately half of what had been observed in historical control group (RR 0.521, 95% CI 0.297-0.916; *p* = 0.0235) and, therefore, the study suggested a possible efficacy of this treatment (Figure 4). The number of NNT to prevent one additional bad outcome was 8.8 (95% CI 4.8-45.9). In addition, newborns treated with propranolol showed a trend toward a reduced progression to stage 2 (RR 0.855, 95% CI 0.715-1.021; *p* = 0.083) and to stage 3 without plus (RR 0.724, 95% CI 0.491-1.069; *p* = 0.104). Consequently, the number of treatments with laser or bevacizumab was significantly reduced by propranolol eye micro-drops (RR 0.468, 95% CI 0.242-0.905; *p* = 0.024 and RR 0.381, 95% CI 0.118-1.230; *p* = 0.107, respectively). Nine of them received laser therapy and three received anti-VEGF injections. No newborns progressed to stage 4 or 5, and no newborn was treated with vitrectomy or cryotherapy, but the number of patients in these stages was also too small in the historical group to obtain statistically meaningful results (Table 3).

**Table 3:** Ophthalmologic outcome.

**ROP progression**	**Treated group**	**Historical control group**	***p***	**Risk ratio (95% CI)**
Newborns*, n*	97	333		
Stage 2, *n* (%)	58 (59.8)	233 (70)	0.083	0.855 (0.715–1.021)
Stage 3, *n* (%)	23 (23.7)	109 (32.7)	0.104	0.724 (0.491–1.069)
Stage 2 or 3 ROP with plus, *n* (%)	12 (12.4)	79 (23.7)	0.023	0.521 (0.297–0.916)
Stage 4 ROP, *n* (%)	0	4 (1.2)	0.513	0.379 (0.021–6.973)
Stage 5 ROP, *n* (%)	0	1 (0.3)	0.938	1.136 (0.047–27.669)
Treatment with laser photocoagulation, *n* (%)	9 (9.3)	66 (19.8)	0.024	0.468 (0.242–0.905)
Treatment with bevacizumab, *n* (%)	3 (3.1)	27 (8.1)	0.107	0.381 (0.118–1.230)
Vitrectomy, *n* (%)	0	4 (1.2)	0.513	0.379 (0.021–6.973)
Cryotherapy, *n* (%)	0	1 (0.3)	0.938	1.136 (0.047–27.669)

The six newborns who progressed to stage 2 plus were treated with laser or anti-VEGF earlier than those six that progressed to stage 3 plus (21 ± 9 vs. 27 ± 8 days; *p* = 0.291). The mean age and the birthweight of newborns who needed laser or anti-VEGF treatment were significantly lower when compared with the overall population of the study (25.3 ± 1.2 vs. 26.7 ± 2.0 weeks, *p* = 0.0019; 702 ± 142 vs. 877 ± 250 g, *p* = 0.0192), confirming the importance of these two risk factors. Failed newborns also showed a higher incidence of co-morbidities (increased incidence of intestinal perforation, sepsis, intra-ventricular hemorrhage), but statistical significance was observed exclusively for a higher incidence of surgical patent ductus arteriosus (58.3% vs. 18.4%, *p* = 0.0017).

Finally, 37 newborns continued propranolol treatment for 90 days because the retinal vascularization had not completed, but no rebound was reported when treatment was discontinued.

Unexpectedly, all three patients treated for cardiac diseases with oral propranolol before the appearance of ROP who then enrolled after ROP stage 1 development, were then unresponsive to propranolol eye micro-drops and needed laser or anti-VEGF treatment.

## Discussion

This trial, using the Simon two-stage optimal design, showed that propranolol 0.2% eye micro-drops are safe and probably effective in reducing the expected progression of ROP from stage 1 to stage 2 plus or 3 plus. In our historical group, this progression occurred in 23.7% of the infants, a value in line with incidence reported in the ETROP Cooperative Group study (25) and additional studies (26), corresponding to the 2.9% of all VLBW admitted, and in line with incidence recently reported in UK (27), while topic ocular propranolol reduced this progression rate to 12.4% (corresponding to the 1.1% of all VLBW admitted). The mean plasma propranolol levels were consistently below the cut-off value of 20 ng/mL, and the biochemical, hematologic, hemodynamic, and respiratory parameters in the treated newborns were reassuring. No adverse effects and no increase in hospitalization were observed after topical administration of propranolol 0.2%. Micro-drops were also well-tolerated locally, without any signs of topical injury, despite the treatment being started earlier and at double the concentration of the one used in our previous study (16). No rebound was reported, probably because 90 days as an extreme term of treatment was an acceptable limit.

Comparing the results of this trial with the previous one (16), we can conclude that by increasing the dose from 0.1 to 0.2%, we reduced the progression of ROP by 50%, without adverse effects and with low plasma concentration. However, the optimal dosage and concentration of the propranolol eye micro-drops are still uncertain. Nevertheless, we cannot exclude that an even higher dose might further increase the efficacy of treatment, without compromising safety and tolerability.

One of the most unexpected results of this study was the observation that all 3 newborns treated with oral propranolol before the development of ROP for heart conditions, failed to respond to eye micro-drops treatment. A fourth newborn, who had been treated with oral propranolol in the first weeks of life, before ROP appearance, but had not been enrolled in the trial because a stage 2 ROP was detected in the first examination, developed a dramatically aggressive ROP (bilateral stage 4) despite laser treatment and vitrectomy. In the study protocol, no exclusions had been foreseen for newborns receiving propranolol prior to the occurrence of ROP. Therefore, the enrolment of these newborns was inevitable. However, this finding deserves some speculation.

This probable protective effect of propranolol during the proliferative phase together with a probable deleterious effect during the ischemic phase are compatible with the bi-phasic and “specular” pathogenesis of ROP and the role of the β-adrenergic system in the different stages of the disease. It is plausible, in fact, that the administration of propranolol, a molecule able to reduce the production of VEGF, may be effective during the proliferative phase of ROP, when VEGF is dramatically increased and a reduction of VEGF is required, but detrimental if administered during the ischemic phase, when levels of VEGF are too low to permit a normal vascularization of the retina and an increase of VEGF is suitable. This apparent contradictory effect has in fact already been described for other molecules, such as VEGF or NO, which may be useful if administered during the ischemic phase, but harmful during the proliferative phase (28–31). Based on our results, we speculate that propranolol is only useful during the proliferative phase of ROP, while we cannot exclude that during the ischemic phase the agonism of β-AR might prevent or reduce the retinal vaso-obliteration, thanks to the ability of the β-AR associated increase of VEGF. In fact, β-AR agonists have been shown to ameliorate the retinal damage in experimental models of diabetic retinopathy, preventing the degeneration of retinal capillaries (32, 33). Nevertheless, in contrast with our data on the possible detrimental effects of early exposure to propranolol on ROP development, Sanghvi et al. recently investigated, in a randomized controlled trial, the early use of low-dose oral propranolol for the prevention of ROP. Propranolol started on the 7th day of life and maintained up to a corrected GA of 37 weeks or complete retinal vascularization did not aggravate ROP and they even observed a non-statistically significant trend in ROP reduction (34). Altogether, the current data warrants further pre-clinical investigations to confirm our results suggesting a protective role of β-ARs during the ischemic phase of ROP. Our hypothesis that oxygen-induced vaso-obliteration in response to hyperoxia occurs through β-ARs down-regulation, which could in turn be exacerbated by the blockade of β-ARs, needs to be further explored in animal models.

Regarding the optimal time to start the eye drop treatment, it is likely that the early start of propranolol administration at stage 1 ROP may have contributed to the greater effectiveness if compared to the previous 0.1% trial (16). However, the negative outcome in newborns treated with propranolol during the “ischemic phase” of ROP suggests that propranolol cannot be considered as a preventive treatment for ROP, but rather a risk factor for a more aggressive ROP. Therefore, the only possible strategy to increase the efficacy of propranolol topical treatment might be to increase the dose and not to start the treatment before ROP appearance.

Although this study suggests that topical administration of propranolol 0.2% eye micro-drops is an effective therapeutic approach to counteracting ROP progression, some limitations have to be discussed.

### Limitations

The design of the trial, based on a comparison with a historical group and without a prospective control group, implies that this study basically has an exploratory purpose, and does not allow for definitive conclusions to be drawn. For example, the perinatal characteristics and the clinical management of the two compared populations were not perfectly homogeneous (as demonstrated by the differences in the Apgar score and the different incidence rate in surfactant administration and oxygen exposure). We cannot exclude that these differences may have impacted ROP occurrence and progression, although it is difficult to assess in which direction. Moreover, we cannot exclude that laser or anti-VEGF treatments have always been appropriate, considering that sometimes a number of infants were treated with a clinical diagnosis milder than stage 2 or 3 plus ROP (35). Therefore, only a prospective placebo-controlled study can avoid bias arising, respectively, from non-homogeneous groups or uneven intervention criterion for laser photocoagulation or anti-VEGF treatment.

We observed a high variability in plasma propranolol concentration. The considerable inter-subject variability had been previously observed, probably because of the variability in absorption and metabolism (23). However, in this study, many other biases could have influenced this variability. The compression of the lacrimal sac following the application of each micro-drop can have variable efficacy between patients. Moreover, adherence or non-adherence to the correct compression of the lacrimal ducts has not been thoroughly evaluated in this study, and this point should be well-analyzed in future studies. Moreover, the delivery system is a pipette that is not specifically designed for the current purpose and nurses/parents have learned to use it in a short time. So, we cannot exclude that the high variability might also be due to the inaccurate administration of the eye micro-drops. Interestingly, 3 of the 12 failed patients showed plasma propranolol values under the detection limit of the analytical system (<2.5 ng/mL) (22). Therefore, it is likely that a more reliable delivery system could guarantee a more constant concentration of the drug and probably better results.

Propranolol is a non-selective β-AR antagonist, predominantly active on β1 and β2-ARs. Recent findings demonstrated that β3-ARs are also actively involved in hypoxia-induced retinal neo-vascularisation (36, 37). It is possible that in the near future, the antagonism of β-ARs might also include β3-AR blockers, but unfortunately, currently there are no approved blockers for targeting this receptor in humans.

## Conclusion

In conclusion, this second phase IIB study shows that propranolol 0.2% eye micro-drops reduced ROP progression when administered during the proliferative phase of the disease. Propranolol treatment was well-tolerated even in preterm newborns with a high prevalence of co-morbidities. Therefore, subsequent confirmations from randomized placebo-controlled trials are definitely warranted.

## Ethics Statement

This study was carried out in accordance with the recommendations of Italian Medicines Agency Institutional Review Board and the Ethics Committees of all the participating centers with written informed consent from all subjects. All subjects gave written informed consent in accordance with the Declaration of Helsinki. The protocol was approved by the name of committee.

## Author Contributions

LF and GiC conceptualized and designed the study, drafted the initial manuscript, and reviewed and revised the manuscript. EB, LP, GA, GiR, GeR, VB, PT, BT, AM, GBu, MA, AB, GaC, SA, and DN contributed to patients' enrollment, neonatal monitoring, and acquisition of data. PF, SO, BC, MS, MV, GBo, SD, GN, and SP contributed to patients' enrollment, contributed to ophthalmologic evaluations and acquisition of data. GM and GF were responsible for propranolol measurements, contributed to the drafting the manuscript. SM, IC, and MC contributed to the analysis of the data, had primary responsibility for statistical analysis. PB and MD contributed to conception and design of the study, contributed to the drafting the manuscript. AC was responsible for propranolol eye drops preparation and distribution to all the centers. AP, member of Clinical Trial Office of Meyer Hospital of Florence, coordinated the ethics committees of the seven centers participating to the trial and was responsible for relations with the Italian Medicines Agency EV, GD, and FM contributed to conception and design of the study, supervised the design and the execution of the study, critically reviewed the manuscript for important intellectual content. All authors approved the final manuscript as submitted and agree to be accountable for all aspects of the work.

### Conflict of Interest Statement

The authors declare that the research was conducted in the absence of any commercial or financial relationships that could be construed as a potential conflict of interest.

## Abbreviations

ROP: Retinopathy of Prematurity
GA: Gestational Age
OIR: Oxygen-induced Retinopathy
β-AR: β-adrenoreceptor
HIF: Hypoxia-Induced Factor
VEGF: Vascular Endothelial Growth Factor
IGF-1: Insulin Growth Factor-1
NICU: Neonatal Intensive Care Units
AP-ROP: Aggressive Posterior ROP
ETROP: Early Treatment for Retinopathy of Prematurity
CV: Coefficient of Variation.
